# Robustness of Frequency Division Technique for Online Myoelectric Pattern Recognition against Contraction-Level Variation

**DOI:** 10.3389/fbioe.2017.00003

**Published:** 2017-02-06

**Authors:** Bahareh Tolooshams, Ning Jiang

**Affiliations:** ^1^Department of Electrical and Computer Engineering, University of Waterloo, Waterloo, ON, Canada; ^2^Department of Systems Design Engineering, University of Waterloo, Waterloo, ON, Canada

**Keywords:** electromyography, robustness, online performance, myoelectric control, muscle contraction level

## Abstract

Contraction-level invariant surface electromyography pattern recognition introduces the decrease of training time and decreases the limitation of clinical prostheses. This study intended to examine whether a signal pre-processing method named frequency division technique (FDT) for online myoelectric pattern recognition classification is robust against contraction-level variation, and whether this pre-processing method has an advantage over traditional time-domain pattern recognition techniques even in the absence of muscle contraction-level variation. Eight healthy and naïve subjects performed wrist contractions during two degrees of freedom goal-oriented tasks, divided in three groups of *type I, type II*, and *type III*. The performance of these tasks, when the two different methods were used, was quantified by completion rate, completion time, throughput, efficiency, and overshoot. The traditional and the FDT method were compared in four runs, using combinations of normal or high muscle contraction level, and the traditional method or FDT. The results indicated that FDT had an advantage over traditional methods in the tested real-time myoelectric control tasks. FDT had a much better median completion rate of tasks (95%) compared to the traditional method (77.5%) among non-perfect runs, and the variability in FDT was strikingly smaller than the traditional method (*p* < 0.001). Moreover, the FDT method outperformed the traditional method in case of contraction-level variation between the training and online control phases (*p* = 0. 005 for throughput in *type I* tasks with normal contraction level, *p* = 0.006 for throughput in *type II* tasks, and *p* = 0.001 for efficiency with normal contraction level of all task types). This study shows that FDT provides advantages in online myoelectric control as it introduces robustness over contraction-level variations.

## 1 Introduction

Surface electromyography (sEMG) signals, the muscles’ electrical activities recorded at the skin surface, are used for the control of multifunction upper-limb prostheses (Parker et al., 2006). Currently, with one recently emerged exception, the techniques used in commercial prostheses provide very limited functionalities which can only be operated over no more than two degrees of freedom (DoF) in a sequential manner with unintuitive switching commands, such as a strong contraction over all EMG channels. One of the techniques used to improve the performance of prosthetic control over the past decades is pattern recognition (Scheme and Englehart, 2011), which is the basis of a recently commercial product, COAPT’s complete control system.^1^

For one-to-one mapping of EMG signals to specific contraction types, the pattern of surface electromyography (sEMG) elicited during specific movements are stored and analyzed by the pattern recognition algorithm in the training phase; then, the trained classifier is used to classify the EMG signal during the control phase into the intended movement (Li et al., 2010). To achieve low classification error, the signals or features extracted from the signals, in the control phase should be stationary, or similar to those in the training phase. Currently in the literature, various factors that can induce non-stationary change to EMG and EMG features between the training and control phases have been identified. Consequently, these non-stationarities can significantly affect the performance of pattern recognition-based (PR-based) algorithms in activities of daily living (ADL). This lack of robustness is indeed one of the major obstacles for the pattern recognition algorithms to be implemented in commercial products (Jiang et al., 2012). These non-stationary factors include, but are not limited to, arm and trunk positions (Fougner et al., 2011), electrode shifts (Young et al., 2012), subject learning (He et al., 2015a), and contract levels (Kaufmann et al., 2010). Specifically, muscle contraction level was shown to be one of the factors inducing non-stationarity between the training phase and the actual control phase (Kaufmann et al., 2010). The authors showed that when the contraction level between the training and control phases differed, the performance of a large number of classifiers, which had been previously reported in myoelectric control, dropped significantly. In order to address this type of degradation, these classifiers have to be trained with a large range of contraction levels. However, this approach can be impractical in real-world applications, particularly when there are a large number of classes. Alternatively, features of sEMG or specific portions of sEMG signal that do not change (or have limited change) at different contraction levels is preferable as they can lead to an elegant control scheme that is inherently robust against varying contraction levels between the training and control phases. In He et al. (2015b), a frequency-based feature set is proposed as the first attempt to realize a contraction-level independent myoelectric pattern recognition classification. It was shown that with this new feature set, the classification performance was significantly better than a classic pattern recognition algorithm when presented with contraction levels not seen in the training phase. More recently, Al-Timemy et al. (2015) also proposed a feature set based on spectral moment descriptors to improve the robustness of myoelectric control with the presence of contraction-level variation. However, both of these studies (Al-Timemy et al., 2015; He et al., 2015b) were offline studies, where the advantage may or may not be able to translate to online controllability of the prostheses (Jiang et al., 2014c). Lock et al. (2005) showed that the offline performance of PR-based algorithms was not correlated with its online performance. By comparing three simultaneous and proportional myoelectric control algorithms of non-negative matrix factorization (NMF), linear regression (LR), and artificial neural networks (ANN), Jiang et al. (2014c) showed that the offline performance of the algorithms is weakly correlated to the online performance control. In this sense, the advantage of a feature set, or a signal pre-processing technique for online myoelectric control, is not known from the offline study until it is examined in an online myoelectric control scheme.

More importantly, neither of these two studies (Al-Timemy et al., 2015; He et al., 2015b) explicitly exploited the advantage of the fundamental properties of motor units involved in the generation of sEMG signals. Based on the Henneman’s size principle, the recruitment of motor units at different levels is ordered such that smaller units with lower firing rates are recruited at low contraction levels, and larger units with higher firing rates are recruited progressively at higher contraction levels (Henneman et al., 1965). Indeed, the differences of firing rates of active motor units at different contraction levels can lead to different characteristics among different frequency bands of sEMG. In addition, not only does the power spectrum density function (PSDF) of EMG not change uniformly over all the frequency bands in the case of varying muscle contraction levels but also some frequency bands have less dependency on contraction level (Roman-Liu and Konarska, 2009). As such, extracting frequency band-specific information is beneficial in identifying the changes at different frequency bands and in increasing the robustness of the algorithm against changing contraction levels.

The purpose of this study was to investigate the effect of FDT in an online myoelectric control scheme as the applied contraction level varies in the training and control phases. The online control phase was achieved through goal-oriented tasks. The current study explored the normal and high muscle contraction levels, as they are more likely to be used during wrist contractions.

## 2 Methods

### 2.1 Subjects

This study was conducted with eight healthy subjects (4 males, 4 females, 18–54 years old), denoted by SUB01–SUB08. None of the subjects had prior experience with EMG or myoelectric control systems before the experiment.

### 2.2 Algorithm

The sEMG signals captured from the subjects were processed with the following steps. First, in order to remove the background noise, particularly 60 Hz line interferences, in the acquired sEMG signals, the method of Common Averaging was used. In this method, the mean of the signal from all seven channels was calculated and subtracted from each individual channel. Following the common average step, two filtering methods were used, namely bandpass and frequency division technique (FDT). For bandpass, the sEMG signals were bandpass filtered (second order, Butterworth) from 20 to 450 Hz. In the literature, this is the most commonly used filtering method for myoelectric control (Scheme and Englehart, 2011). For FDT, the aim was to divide the signals into several channels containing specific frequency bands in order to achieve the advantage of sub-bands, which have low dependency on contraction level. In this method, the signals were filtered by a bank of filters (second order, Butterworth) with frequency bands of 20–92, 92–163, 163–235, 235–307, 307–378, and 378–450 Hz; these ranges of frequencies were chosen from He et al. (2015b). Hence, the output of FDT had a dimension of 42, as opposed to the 7-dimension bandpass filter output.

Following the filtering operation, the classic time-domain features (Hudgins et al., 1993) were extracted from the output of the two filtering schemes. Principal component analysis (PCA) was used to reduce the dimension of the feature space: components containing 95% of the variance of the overall features were retained. In this step, the bandpass and FDT method could result in a different number of PCA components. Subsequently, linear discriminant analysis (LDA) was used for classification. The choice to combine time-domain features and LDA classifier was shown to be the optimal processing method in PR-based myoelectric control literature, particularly in real-time control studies (Scheme and Englehart, 2011). The online myoelectric control experiment of the current study is described in detail next.

### 2.3 Experiment Protocol

During the experiment sessions, the subject placed his/her dominant arm at the side of the body in the neutral position and seated comfortably at a chair. A computer screen was placed in front of the subject at a distance of approximately 1 m. Seven surface electrodes (H124SG, Foam Hydrogel, Covidien) were placed equidistantly along the forearm at approximately 2/3 of the forearm length from the wrist. A high-accuracy bio-signal amplifier (g.USBamp, g.Tec Medical Engineering, Austria) at the sampling rate of 1200 Hz with 24 bit A/D was used for the data acquisition of sEMG signals. For each subject, the experiment protocol consisted of two sessions. The sessions were similar and took place at least two days, approximately three days apart. The first session was a familiarization session because all subjects were naïve to EMG and myoelectric control. This familiarization session was also used to minimize the failure rate of tasks in all the runs, so as to minimize the bias in subsequent statistical analysis. Each session consisted of two phases: a training phase and a control phase, as described in detail in Sections 2.3.1 and 2.3.2. During the control phase, the subject would perform goal-oriented tasks (as described in Section 2.3.2).

#### 2.3.1 Training Phase

Following on-screen instructions, the subject performed a series of wrist contractions that would activate two wrist DoF: wrist flexion/extension and wrist pronation/supination. Two sets of data were collected for algorithm training/calibration purposes: one for wrist movements with normal contraction level of the forearm muscles, at the comfort of the subject (labeled as *train-normal* data); another one for wrist movements with high contraction level of the forearm muscles, for which the subject intended to exert as much effort as possible (labeled as *train-high* data). *Train-normal* or *train-high* data were used for training/calibration of the LDA algorithm (see Section 2.2) depending on the run in the control phase.

#### 2.3.2 Control Phase

**Figure 1:**
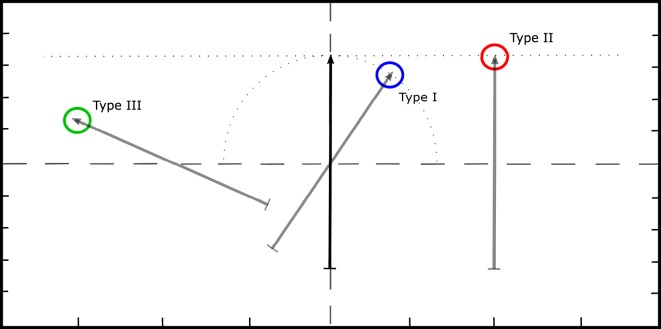
**Goal-oriented tasks performed by the subjects**. The black arrow shows its position at the beginning of each task. The gray arrows represent the desired position for *type I, type II*, and *type III* targets.

This phase would start immediately after the two LDA classifiers were trained: one for bandpass and one for FDT. In this phase, four experimental runs were performed. For each run, one of the two filtering techniques, bandpass or FDT, was used to filter the raw sEMG data, which were acquired when the subject performed wrist contractions (flexion/extension or supination/pronation). The real-time EMG processing window was 150 ms, and the LDA produced a classification decision every 50 ms. No majority vote was implemented. The classification outcome of the LDA, i.e., one of the five classes of flexion, extension, supination, pronation, and no-action, was used to control the following movements of an on-screen arrow: left, right, clockwise rotation, counter-clockwise rotation, and no-movement, respectively (for left-handed subjects, the directions of movements in the two DoFs were reversed). Thus, by performing wrist contractions, the subject was able to control the movement of the arrow in real time. The tasks for the subject were to move the arrow so that the tip of the arrow could hit various circular targets with relative area of 1.4% with respect to the working space (see Figure 1). For the task to be counted as successful, the tip of the arrow must stay inside the target for at least 300 ms within a 20-s interval. The task was labeled as a failure if the subject did not reach the goal within 20 s. Hence, the subjects were instructed to complete the tasks as fast as they can. Four runs of this phase were combinations of the filtering methods (see Section 2.2) and the training conditions (see Section 2.3.1). *Normal-bandpass* was the run where the algorithm was trained with *train-normal* data, and the bandpass filtering method was used on the sEMG signals. In the *normal-FDT* run, the algorithm was trained with *train-normal* data, and the FDT filtering method was used. In the *high-bandpass* and *high-FDT* runs, the control algorithm was trained with *train-high* data, and the filtering method of bandpass and FDT were used, respectively. The order of the runs was randomly chosen for each subject. In each run, 60 targets located at different places on the screen were presented to the subject. Three types of targets, each containing 20 targets, were shown to the subjects. For *type I* targets, ideally the subject would only need to perform wrist supination or pronation to reach the target. For *type II* targets, either wrist flexion or extension was needed to reach the target. For *type III* targets, the subject should perform both *type I* and *type II* functions to reach the target. For all the subjects, the targets were shown with the order of *type I, type II*, and *type III* targets.

### 2.4 Performance Analysis

To analyze real-time control performance in the goal-oriented tasks, the time taken to reach the goal successfully and the trajectory of the tip of the arrow were recorded. Using the recorded measures, five performance indices were calculated: time to reach (*T2R*), throughput (*TP*), path efficiency (Γ), near miss (*NM*), and completion rate (*CR*) as defined in Jiang et al. (2014c).

#### 2.4.1 Time to Reach [*T2R* (s)]

*T2R* was the time taken by the subject to reach the goal. *T2R* would be 20 s if the subject was not able to complete the task within 20 s.

#### 2.4.2 Throughput [*TP* (bit/s)]

The performance index of *TP* quantified how much information could be delivered by the subject through the control movements within the duration the task. This index was calculated as in Jiang et al. (2014c), by the ratio of the task difficulty and extitT2R.

#### 2.4.3 Path Efficiency [Γ (%)]

The length of the optimal path from the zero position to the position of the target was compared to the length of the path covered by the subject in each task. The ratio of these two was labeled as Γ.

#### 2.4.4 Near Miss [*NM* (k)]

Near miss performance index was an overshoot factor which measured the number of times the tip entered the circle of target but exited the circle in less than 300 ms.

#### 2.4.5 Completion Rate [*CR* (%)]

*CR* was the ratio between the number of successful tasks and performed tasks. 20 trials performed by each subject in a specific scenario were used to calculate the completion rate for that specific subject in the scenario.

### 2.5 Statistics

The main hypothesis of the study is that compared to bandpass, FDT provides a more accurate online performance with the PR-based myoelectric control paradigm, and the secondary hypothesis is that FDT is more robust against the contraction level, with which the algorithm is trained. In order to test these hypotheses, Kruskal–Wallis and Levene tests were performed to investigate if the *CR* of the process methods (bandpass, FDT) were on average different, and if the variability of the *CR* was different, respectively. To avoid the entire dataset being dominated by runs with 100%, only non-perfect runs (*CR* lower than 100%) were analyzed. Following these non-parametric tests, repeated-measure analysis of variance (ANOVA) tests were performed for the first four performance indices from successful trials, with subject (SUB01–SUB08) as the factor measures were repeated on. The two-way interactions of the other three factors: target type (*type I, type II, type III*), training method (*train-normal, train-high*), and process method (bandpass, FDT), were included in the initial ANOVA. For cases where there was no significant two-way interaction, the interactions were removed from the analysis. In case of significant two-way interactions, the level of one of the interacting factors was fixed, and the focused ANOVA was performed. As the process method of FDT was the interest of this study, the target type and training method was fixed whenever possible. For the analysis of each performance metric, the trials with standardized residuals equal or larger than four were removed from the analysis. In case of significance on the main factors, the Tukey comparison was conducted. For all the tests, the level of significance was 0.05.

## 3 Results

**Figure 2:**
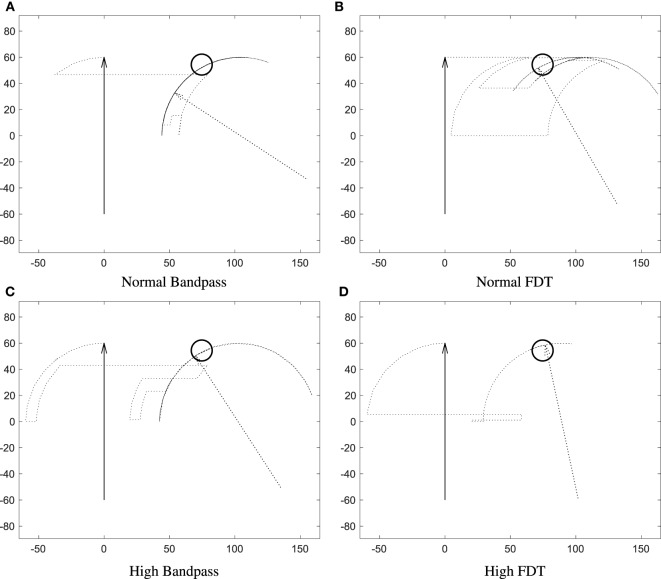
**Representative trajectories of the goal-oriented task (*type III* target) for *normal-bandpass, normal-FDT, high-bandpass*, and *high-FDT***. The trajectory data were from SUB07. For **(A)**, the subject was not able to complete the task successfully within 20 s. For **(B)**, the *T2R, TP*, Γ, and *NM* were 16.40 s, 0.41 bit/s, 7.36%, and 6, respectively. For **(C)**, the *T2R, TP*, Γ, and *NM* were 15.39 s, 0.29 bit/s, 8.03%, and 3, respectively. For **(D)**, the *T2R, TP*, Γ, and *NM* were 9.90 s, 0.45 bit/s, 17.9%, and 2, respectively. Arbitrary units for all axes.

**Figure 3:**
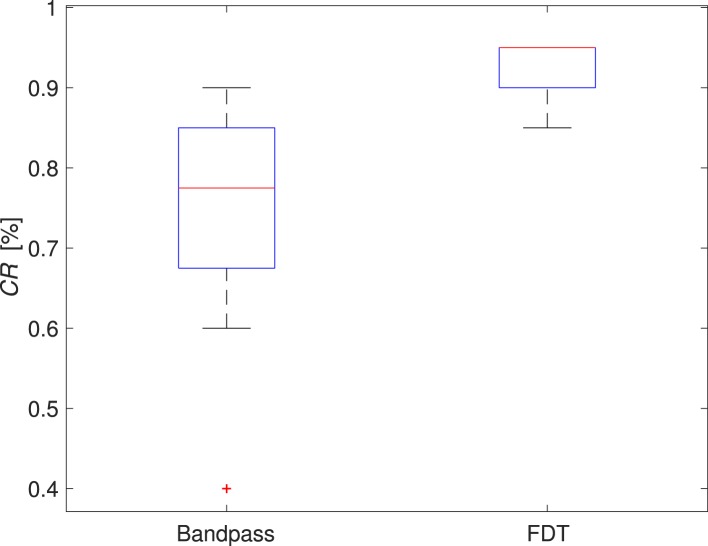
**Completion rate of all non-perfect runs**.

The trajectories of goal-oriented tasks, from one subject for the combinations of process and training methods, are presented in Figure 2. As shown in the figure, the performance of the runs using FDT was better than the performance of the runs with the bandpass method. The median *CR* of runs with the FDT method was 95% across all non-perfect runs and was 77.5% with the bandpass method. More importantly, the variability in *CR* for FDT was 3.45%, which was strikingly smaller than the bandpass method with the variability of 12.87% (*p* < 0.001). In fact, the *CR* of 50% of the FDT runs lies between 90 and 95%, whereas for bandpass it lies between 66.25 and 85%. Subsequent statistical analysis supported this observation, indicating FDT provided a clear advantage in real-time control over bandpass such that the participants were able to complete the tasks more successfully. Figure 3 clearly presents that the *CR* of the non-perfect runs are higher with lower variability when the FDT method was used. For the successful trials, in case of found significance, the other performance indices are summarized below. For *type II* targets, when *train-high* data were used, the *T2R* of the FDT method was 3.26 s, which was lower than the bandpass method with a *T2R* of 4.16 s. The mean of *TP* for *type II* targets with the FDT method was 1.64 bit/s, while with the bandpass method it was only 1.41 bit/s. The FDT method outperformed the bandpass method in throughput (*TP* = 2.10 vs. 1.73 bit/s) for *type I* targets in *train-normal* runs. In *train-normal* runs, the Γ with FDT and bandpass were 58.23 and 51.46%, respectively.

**Figure 4:**
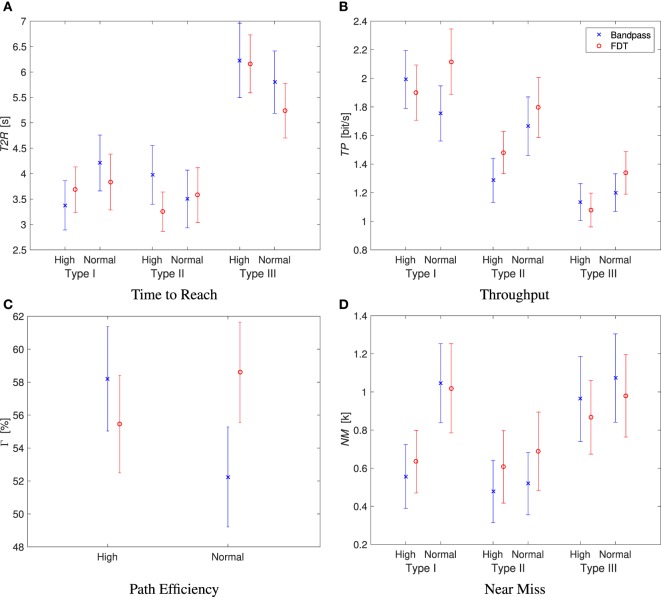
**Summary of the performance indices of *T2R, TP*, Γ, and *NM***. The 95% confidence interval for mean is shown here. The outliers are removed from the analysis. **(A)** Time to reach. **(B)** Throughput. **(C)** Path efficiency. **(D)** Near miss.

There were significant two-way interactions between the target type and training method for *T2R, TP*, and *NM* with corresponding *p*-values of 0.002, 0.016, and 0.014, respectively, and also between the process and training methods with *p*-value of 0.003 for Γ. Therefore, a series of focused ANOVA were performed, which are summarized in Table 1. For *T2R*, a focused ANOVA was performed with fixed target type. Only for *type II* targets with *train-high* runs, the *T2R* of FDT was found to be statistically better than the *T2R* of bandpass (*p* = 0.003). For *type I* and *type III* targets, no significance between the two process methods (*p* = 0.681 and *p* = 0.085, respectively) was found. For *TP*, the focused ANOVA with only *type I* targets found significant interaction in *train-low* runs between the two main factors of process and training methods (p = 0.005). In this case, no significance between the two process methods (*p* = 0.695) was found using *train-high* data. For *type II* targets, FDT was found to be statistically better than bandpass (*p* = 0.006). The *TP* of runs with FDT and bandpass was not found to be statistically different for *type III* targets (*p* = 0.469). For Γ, a focused ANOVA was performed, and the test found that FDT and bandpass were statistically different in all *train-normal* runs. In this case, a Tukey comparison revealed that FDT resulted in significantly higher path efficiency than bandpass with a *p*-value of 0.001. For *NM*, in neither of the target types (*type I, type II*, and *type III*), was significance found (*p* = 0.996, *p* = 0.841, and *p* = 0.195, respectively). Figure 4 presents the result of the statistical analysis for the first four performance indices.

**Table 1:** **Summary of the statistical analysis for the comparison of FDT and bandpass in performance indices**.

Performance index			T2R		TP		Γ	NM
Focused two-way ANOVA	Target type	I	*p* = 0.681		Normal	*p* = 0.005 *FDT* > *BP*	N/A	*p* = 0.996
					High	*p* = 0.695		
		II	Normal	*p* = 0.517	*p* = 0.006 *FDT* > *BP*			*p* = 0.841
			High	*p* = 0.003 *FDT* < *BP*				
		III	*p* = 0.085		*p* = 0.469			*p* = 0.195
Focused one-way ANOVA	Training method	Normal	N/A		N/A		*p* = 0.001 *FDT* > *BP*	N/A
		High					*p* = 0.666	

*In case of found significance, the table presents which process method was better*.

*BP = bandpass*.

In summary, for the key performance indices of *TP, T2R*, and Γ, FDT outperformed for at least one of the target types. For *train-normal* runs, FDT resulted in higher *TP* for *type I* and *type II* targets, and it performed with higher Γ for all target types. In other words, the advantage of FDT was more pronounced when the contraction level of the training data differed from the contraction level of the real-time control tasks; for *train-normal* runs, FDT had a tendency to have better performance than bandpass. This result was due to the high muscle contraction that the subjects applied during the runs as they were asked to complete the trials as fast as they can. Consequently, the muscle contraction level used during the real-time trials was closer to *train-high* trials than *train-normal* trials. The outperformance of FDT over bandpass, while the contraction level of the training data and real-time control were different, clearly highlights the robustness of the FDT method against the contraction level; and this result is in agreement with the secondary hypothesis. The comparison of the process methods revealed that FDT has advantage over bandpass. Also, this advantage was supported by the significantly lower *CR* variability that FDT had with respect to bandpass. This low variability in *CR* for FDT supported the main hypothesis of this experiment, which was that FDT provides better online performance compared to bandpass. It is important to note that these comparisons were performed only with successful trials (failure trials were only used for performance index of *CR*). These analyses implicitly penalized FDT, which had a significantly higher *CR*. Because if the maximal allowed task completion time was set at a longer time, more trials with bandpass with longer completion time, worse path efficiency, and more overshoot would have been included.

## 4 Discussion

In this study, two sEMG signal pre-processing methods, namely bandpass and FDT, were compared to investigate their robustness against varying contraction levels and consequently the accuracy of PR-based myoelectric control algorithms in the presence of varying contraction levels. A goal-directed motor task involving wrist contractions were used, similar to several previous studies (Ameri et al., 2014a,b; Jiang et al., 2014a,b,c) of multi-DoF real-time myoelectric control. Five performance indices of a goal-oriented online control tasks, time to reach (*T2R*), throughput (*TP*), path efficiency (Γ), near miss (*NM*), and completion rate (*CR*), were used in this study. It was previously reported that the performance of pattern recognition algorithms is seriously affected by varying muscle contraction levels (Kaufmann et al., 2010; He et al., 2015b). This was confirmed by our results: significantly lower accuracy was observed when the contraction level between the training and control phases was different. Therefore, a more robust signal processing technique that does not lead to online performance degradation with the presence of variation in muscle contraction is highly desirable. In particular, it would be practically appealing to perform the training phase for as few contraction levels as possible.

One of the important outcomes in this study is demonstrating that in an online control scheme, FDT provides a more robust prosthetic control against changing contraction levels than bandpass. Indeed, FDT increases the robustness against contraction level without significantly increasing the computational cost, as only time-domain features are used in the method. The experiment protocol designed for this study intentionally allowed the subjects to have freedom in terms of muscle contraction level in the control phase, as they would in ADLs. This design allowed the subjects to exert high contraction levels during the control phase. As such, the advantage of FDT over bandpass was more pronounced in the *train-normal* runs where the control phase and the training phase data had the most discrepancy in contraction levels. Another reason for this design was to minimize the correlation between the performances of the subjects in the training and control phases. In fact, the subjects were asked to maintain a constant muscle contraction level in the training phases (normal contraction in the normal runs and high contraction in the high runs), and they had no such restriction on the contraction level during the control phases.

Another important outcome of this study is that the performance of PR-based myoelectric control was higher in general when FDT was used in data processing than bandpass. This conclusion comes from the fact that FDT resulted in lower variability in *CR* and also higher *CR* even though the statistical analyses were biased toward the bandpass method by excluding failed trials (as discussed in Section 3).

In this study, similar to Jiang et al. (2014b), the process methods were measured in terms of relative advantage with the performance indices and the control parameters of the tasks (relative area of circular targets, the minimum acceptable time of being inside the circle for success, and the maximum time allowed). To monitor the relative differences, all the subjects’ runs had similar control parameters. Hence, in this paper, although the absolute values of the performance indices were provided, only the statistical difference of the performance indices between the process methods were emphasized.

Considering that all the subjects attended in the study were naïve, the experiment protocol had two sessions for each subject. The first session helped the subjects to become familiar with the protocol of the experiment as well as with using myoelectric control to achieve the goal-oriented tasks.

The main limitation of this study is that the change of feature space is not investigated in the performances. In He et al. (2015b), changes of feature space were studied whenever normalization of data was possible. However, such systematic analysis in feature space change for online performances cannot be conducted as standardization of online data is not possible as in offline analysis.

The current study is only limited to intact-limb subjects. Based on Jiang et al. (2014b), the online performance of PR-based myoelectric control in upper-limb amputees is similar to the performance of intact-limb subjects. Hence, in presence of the amputee subject limitation, it is reasonable to expect a similar advantage of FDT over bandpass in trans-radial amputee subjects. However, this needs to be validated with further experiments.

## 5 Conclusion

In this paper, we analyzed two signal pre-processing techniques for PR-based myoelectric control, aiming at improving system robustness against variation of muscle contraction levels. We investigated the performance of the FDT and bandpass methods in presence of muscle contraction variations between the training and control phases, and we found that FDT significantly outperformed bandpass for PR-based prosthetic myoelectric control, with higher online control performance in general, and a significantly smaller inter-subject variability. More importantly, the online control is significantly more robust against changing contraction levels with the FDT method. FDT can increase the freedom of the subject to vary the muscle contraction level used for control, which provides comfort for the user. To emphasize the advantage of FDT over bandpass, future work would include force/torque measurement, as well as standardized trials in the control phase to systematically study the feature space change with the presence of contraction-level change, as well as validation with trans-radial amputees.

## Ethics Statement

The experiment protocol was approved by the University of Waterloo Research Ethics Committee (#21387). Each subject read and signed the informed consent prior to the experiments.

## Author Contributions

BT contributed to the following: ethical application process, design of the experiment, implementation of the filtering methods, data acquisition through experiment, statistical analysis of the data, drafting the manuscript, and approval of the final version of the manuscript before submission. NJ contributed to the following: ethical application process, concept of the study, design of the experiment, development of the methodology, statistical analysis of the data, revision of the manuscript, and approval of the final version of the manuscript before submission. Both authors, BT and NJ, are responsible for the accuracy of the analysis and conclusions presented in this study.

## Conflict of Interest Statement

The authors declare that the research was conducted in the absence of any commercial or financial relationships that could be construed as a potential conflict of interest.

## Footnotes

^1^https://coaptengineering.com.

## Abbreviations

Γ, path efficiency; *CR*, completion rate; *NM*, near miss; *T2R*, time to reach; *TP*, throughput; ADL, activities of daily living; ANN, artificial neural networks; ANOVA, analysis of variance; DoF, degree of freedom; EMG, electromyography; FDT, frequency division technique; LDA, linear discriminant analysis; LR, linear regression; NMF, non-negative matrix factorization; PCA, principal component analysis; PR-based, pattern recognition-based; PSDF, power spectrum density function; sEMG, surface electromyography.

## References

[B1] Al-Timemy A. Khushaba R. Bugmann G. Escudero J. (2015). Improving the performance against force variation of EMG controlled multifunctional upper-limb prostheses for transradial amputees. IEEE Trans. Neural Syst. Rehabil. Eng. 24, 650–661. 10.1109/TNSRE.2015.2445634 26111399

[B2] Ameri A. Kamavuako E. N. Scheme E. J. Englehart K. B. Parker P. A. (2014a). Support vector regression for improved real-time, simultaneous myoelectric control. IEEE Trans. Neural Syst. Rehabil. Eng. 22, 1198–1209. 10.1109/TNSRE.2014.2323576 24846649

[B3] Ameri A. Scheme E. J. Kamavuako E. N. Englehart K. B. Parker P. A. (2014b). Real-time, simultaneous myoelectric control using force and position-based training paradigms. IEEE Trans. Biomed. Eng. 61, 279–287. 10.1109/TBME.2013.2281595 24058007

[B5] Fougner A. Scheme E. Chan A. D. C. Englehart K. Stavdahl Ø (2011). Resolving the limb position effect in myoelectric pattern recognition. IEEE Trans. Neural Syst. Rehabil. Eng. 19, 644–651. 10.1109/TNSRE.2011.2163529 21846608

[B6] He J. Zhang D. Jiang N. Sheng X. Farina D. Zhu X. (2015a). User adaptation in long-term, open-loop myoelectric training: implications for EMG pattern recognition in prosthesis control. J. Neural Eng. 12, 046005 10.1088/1741-2560/12/4/046005 26028132

[B7] He J. Zhang D. Sheng X. Li S. Zhu X. (2015b). Invariant surface EMG feature against varying contraction level for myoelectric control based on muscle coordination. IEEE J. Biomed. Health Inform. 19, 874–882. 10.1109/JBHI.2014.2330356 25014975

[B8] Henneman E. Somjen G. Carpenter D. O. (1965). Functional significance of cell size in spinal motoneurons. J. Neurophysiol. 28, 560–580. 14328454 10.1152/jn.1965.28.3.560

[B9] Hudgins B. Parker P. Scott R. (1993). A new strategy for multifunction myoelectric control. IEEE Trans. Biomed. Eng. 40. 8468080 10.1109/10.204774

[B10] Jiang N. Dosen S. Muller K. R. Farina D. (2012). Myoelectric control of artificial limbs: is there a need to change focus? [In the spotlight]. IEEE Signal Process. Magazine 29, 150–152. 10.1109/msp.2012.2203480

[B11] Jiang N. Lorrain T. Farina D. (2014a). A state-based, proportional myoelectric control method: online validation and comparison with the clinical state-of-the-art. J. Neuroeng. Rehabil. 11, 110 10.1186/1743-0003-11-110 25012766 PMC4108229

[B12] Jiang N. Rehbaum H. Member S. Vujaklija I. Graimann B. (2014b). Intuitive, online, simultaneous, and proportional myoelectric control over two degrees-of-freedom in upper limb amputees. IEEE Trans. Neural Syst. Rehabil. Eng. 22, 501–510. 10.1109/TNSRE.2013.2278411 23996582

[B13] Jiang N. Vujaklija I. Rehbaum H. Graimann B. Farina D. (2014c). Is accurate mapping of EMG signals on kinematics needed for precise online myoelectric control? IEEE Trans. Neural Syst. Rehabil. Eng. 22, 549–558. 10.1109/TNSRE.2013.2287383 24235278

[B14] Kaufmann P. Englehart K. Platzner M. (2010). “Fluctuating EMG signals: investigating long-term effects of pattern matching algorithms,” in Annual International Conference of the IEEE Engineering in Medicine and Biology (Buenos Aires), 6357–6360. 10.1109/IEMBS.2010.5627288 21096692

[B15] Li G. Schultz A. E. Kuiken T. A. (2010). Quantifying pattern recognition-based myoelectric control of multifunctional transradial prostheses. IEEE Trans. Neural Syst. Rehabil. Eng. 18, 185–192. 10.1109/TNSRE.2009.2039619 20071269 PMC3024915

[B16] Lock B. Englehart K. B. Hudgins B. (2005). “Real-time myoelectric control in a virtual environment to relate usability vs. accuracy,” in Proceedings of the 2005 MyoElectric Controls/Powered Prosthetics Symposium, Fredericton.

[B17] Parker P. Englehart K. Hudgins B. (2006). Myoelectric signal processing for control of powered limb prostheses. J. Electromyogr. Kinesiol. 16, 541–548. 10.1016/j.jelekin.2006.08.006 17045489

[B18] Roman-Liu D. Konarska M. (2009). Characteristics of power spectrum density function of EMG during muscle contraction below 30%MVC. J. Electromyogr. Kinesiol. 19, 864–874. 10.1016/j.jelekin.2008.05.002 18590966

[B19] Scheme E. Englehart K. (2011). Electromyogram pattern recognition for control of powered upper-limb prostheses: state of the art and challenges for clinical use. J. Rehabil. Res. Dev. 48, 643–660. 10.1682/JRRD.2010.09.0177 21938652

[B20] Young A. J. Hargrove L. J. Kuiken T. A. (2012). Improving myoelectric pattern recognition robustness to electrode shift by changing interelectrode distance and electrode configuration. IEEE Trans. Biomed. Eng. 59, 645–652. 10.1109/TBME.2011.2177662 22147289 PMC4234037

